# Composite Properties of Non-Cement Blended Fiber Composites without Alkali Activator

**DOI:** 10.3390/ma13061443

**Published:** 2020-03-22

**Authors:** Wei-Ting Lin, Kae-Long Lin, Kinga Korniejenko, Lukáš Fiala, An Cheng, Jie Chen

**Affiliations:** 1 Department of Civil Engineering, National Ilan University, No.1, Sec. 1, Shennong Rd., I-Lan 260, Taiwan; ancheng@niu.edu.tw (A.C.); jchen@niu.edu.tw (J.C.); 2 Department of Environmental Engineering, National Ilan University, No.1, Sec. 1, Shennong Rd., I-Lan 260, Taiwan; kllin@niu.edu.tw; 3 Institute of Materials Engineering, Faculty of Materials Engineering and Physics, Cracow University of Technology, Warszawska 24, 31-155 Kraków, Poland; kkorniej@gmail.com; 4 Department of Materials Engineering and Chemistry, Faculty of Civil Engineering, Czech Technical University in Prague, Thákurova 7, 166 29 Prague 6, Czech Republic; fialal@fsv.cvut.cz

**Keywords:** fiber reinforced, cementless composites, microscopic property, co-fired fly ash, green materials

## Abstract

The vigorous promotion of reuse and recycling activities in Taiwan has solved a number of problems associated with the treatment of industrial waste. Considerable advances have been made in the conversion of waste materials into usable resources, thereby reducing the space required for waste storage and helping to conserve natural resources. This study examined the use of non-alkali activators to create bonded materials. Our aims were to evaluate the feasibility of using ground-granulated blast-furnace slag (S) and circulating fluidized bed co-fired fly ash (F) as non-cement binding materials and determine the optimal mix proportions (including embedded fibers) with the aim of achieving high dimensional stability and good mechanical properties. Under a fixed water/binder ratio of 0.55, we combined S and F to replace 100% of the cement at S:F ratios of 4:6, 5:5, 6:4. Polypropylene fibers (L/d = 375) were also included in the mix at 0.1%, 0.2% and 0.5% of the volume of all bonded materials. Samples were characterized in terms of flowability, compressive strength, tensile strength, water absorption, shrinkage, x-ray diffraction (XRD) and scanning electron microscope (SEM) analysis. Specimens made with an S:F ratio of 6:4 achieved compressive strength of roughly 30 MPa (at 28 days), which is the 80% the strength of conventional cement-based materials (control specimens). The inclusion of 0.2% fibers in the mix further increased compressive strength to 35 MPa and enhanced composite properties.

## 1. Introduction

Economic development inevitably increases construction activity, which depends heavily on the production of cement. The most direct approach to reduce the CO_2_ emissions associated with the manufacture of cement is to reduce cement consumption or replace cement with other pozzolanic materials (e.g., industrial by-products) with similar binding properties [1,2,3]. Non-cement blended materials can help to reduce construction costs and the negative impact of cement production on the natural environment [4,5]. Considerable advances have been made in the conversion of waste materials into usable resources [6,7], thereby reducing the space required for waste storage and helping to conserve natural resources [8,9].

Researchers have demonstrated that the incorporation of pozzolans as a partial replacement for cement can improve the mechanical properties and durability of the resulting concrete [10,11,12]. Numerous studies have also used pozzolans (fly ash or slag) to entirely replace cement in ordinary concrete or mortar. Many specimens have strength, mechanical properties and durability superior to those of conventional concrete [13,14]. The key factor in achieving a fully hydrated reaction without cement is the inclusion of an alkali activator or the enactment of curing at an elevated temperature. The cementless composites containing alkali activators inevitably increase production costs. However, many industrial by-products can be used as alkali activators to save cost due to its chemical composition with higher alkali contents.

Considerable advances have been made in the low-energy manufacture of non-cement blended materials, particularly in Taiwan, Korea and Japan. Ground-granulated blast-furnace slag can be combined with various supplementary cementitious materials to eliminate the need for alkali activators in the production of materials suitable for civil construction. Many such materials provide high compressive strength (30 MPa to 60 MPa), as well as excellent mechanical properties and durability [15,16,17,18]. The inclusion of polypropylene fibers in composites has been shown to enhance the tensile strength and volume stability of non-cement blended materials [19,20,21]. In recent years, a number of researchers have studied the use of polypropylene fibers or non-cement blended materials; however, there has been little work on the addition of polypropylene fibers to non-cement blended materials. In this study, we combined ground-granulated blast-furnace slag (GGBS) with circulating fluidized bed (CFB) co-fired fly ash and polypropylene fibers to produce non-cement blended materials without alkali activator. Samples were compared with standard Portland cement mortar in terms of mechanical properties, permeability and microstructure. The proposed material is intended to be used as a controlled low-strength material, pervious concrete, reinforced recycled concrete and for other engineering applications [22,23,24].

## 2. Materials and Methods 

### 2.1. Materials

**Figure 1:**
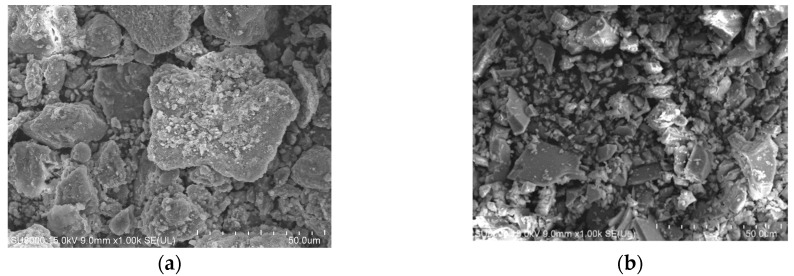
SEM images: (**a**) circulating fluidized bed (CFB) co-fired fly ash and (**b**) ground-granulated blast-furnace slag (GGBS).

**Figure 2:**
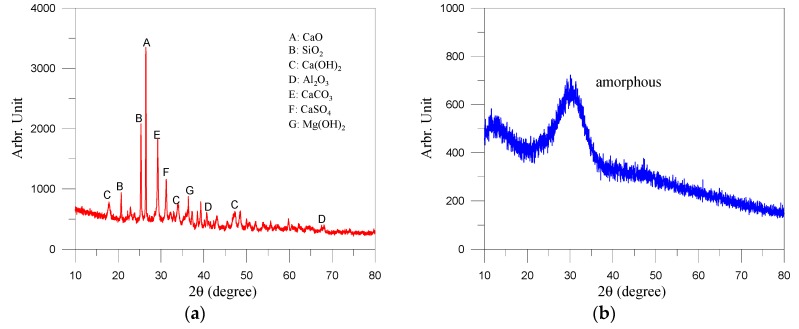
x-ray diffraction (XRD) pattern: (**a**) co-fired fly ash and (**b**) GGBS.

This study used Type I Portland cement with a specific gravity of 3.15 and fineness of 3310 cm^2^/g. The fine aggregate was natural river sand with saturated surface dry (SSD) specific gravity of 2.70, absorption of 1.63% and fineness modulus of 2.33. Hereafter, when used as constituent of non-cement binder, GGBS is referred to as slag (S) and CFB co-fired fly ash is referred to as fly ash (FA). FA was produced in the form of a black powder by Yong Feng Yu, Taiwan with a specific gravity of 2.73. The specific gravity of S was 2.88 and the fineness was 5860 cm^2^/g. The particles of FA passing through a No. 100 sieve (150 μm) are roughly 98% and the fineness was 2800 cm^2^/g. SEM images of the FA revealed irregular polygonal particles similar to those of S as well as rough surfaces, as shown in Figure 1. Figure 2 presents XRD patterns of FA. 

Chemical compositions of FA and S are summarized in Table 1. The chemical composition of FA was analyzed using x-ray fluorescence (XRF) as follows: SiO_2_ (29.47%), Al_2_O_3_ (19.27%) and CaO (35.54%). The polypropylene fibers used in the current study were 12mm in length with an aspect ratio of 375 (d = 32μm) and the fibers were produced by Poplar Co., Ltd. (Taipei, Taiwan). The specific gravity, tensile strength and Young’s modulus of the fibers were 0.91, 250 and 3500 MPa, respectively.

### 2.2. Mix Design and Test Methods

The water/cementitious ratio (w/c) of the mortar specimens was maintained at a constant 0.55, whereas the cementitious materials/fine aggregate mass ratio was 1:2.75, in accordance with ASTM C109 specifications. Table 2 lists the mix design for non-cement blended materials. The specimens were numbered using two number/letters pairs indicating (1) S:F ratio and (2) the percentage of polypropylene fibers. The number after the letter S indicates the percentage of co-fired fly ash (e.g., S40 means 40% fly ash). P refers to ordinary Portland mortar. The last number indicates the percentage of polypropylene fiber (e.g., F1 means 0.1% fiber). In addition, the mixture for a preliminary test of non-cement blended composites was shown in Table 3. The S, FA, fine aggregates and fibers were mixed for about 10 min by using a low speed mixing machine to create the homogeneous mortar specimens and then cast into the metal molds. After demolding, the specimens for standard curing were placed in the standard curing room until the testing age. Table 4 presents the tests performed, the dimensions of the specimens and the standards used in this study. 

## 3. Results and Discussion

### 3.1. Flowability

**Figure 3:**
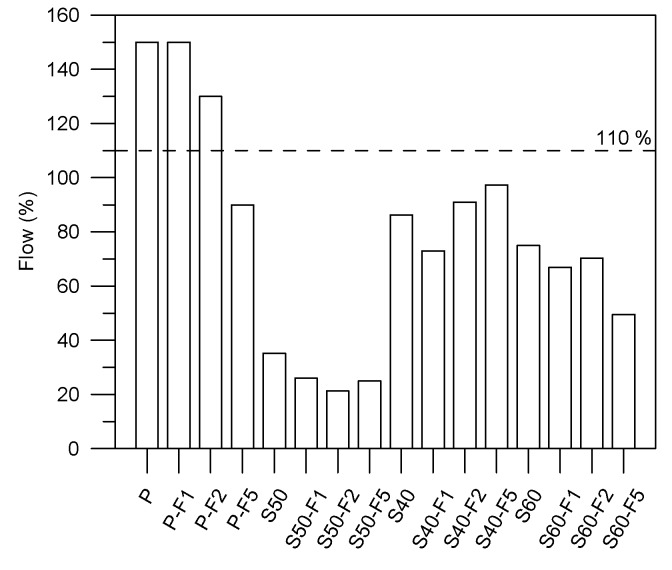
Flow test results.

An increase in the proportion of FA significantly decreases the flowability of the non-cement blended materials as shown in Figure 3. As shown in Table 2, note that suitable mixtures (flow of 110% as shown in Figure 3) were obtained only after adding a water-reducing admixture (superplasticizer) expect for the P series specimens. The 110%-line in Figure 3 indicates the referred flowability in accordance with ASTM C109. For each mixture, the superplasticizer was used to control the workability of the specimens. The results indicated that the specimens containing polypropylene fibers had lower flowability. The amount of superplasticizer required to maintain fluidity was proportional to the amount of FA, due to the high absorbency and angularity of the particles (rough surface as shown in Figure 1). In the cement-based composites, flowability decreased with an increase in the proportion of polypropylene fibers, due to the internal resistance and friction generated through the interaction of fibers. The finding is consistent with the reported in previous studies [25].

### 3.2. Compressive Strength

**Figure 4:**
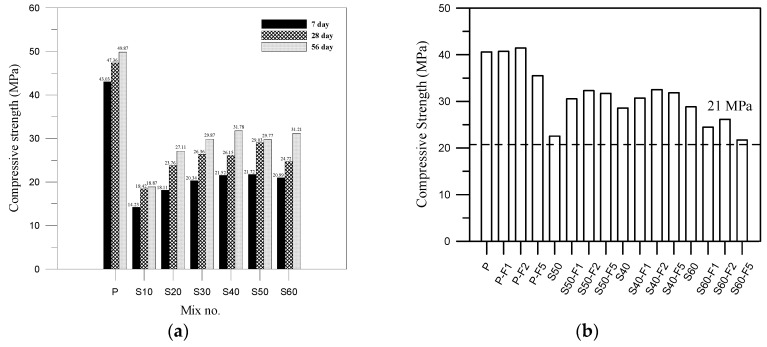
(**a**) Histogram presenting compressive strength results of the preliminary test; and (**b**) compressive strength development at 28 days for all mixes.

Figure 4a presents the preliminary test of the compressive strength of specimens made with non-cement materials at various S/FA ratios without an alkali activator. Compressive strength was shown to increase significantly with curing age between 28 and 56 days. The compressive strength of specimens with 40% FA was significantly higher than that of specimens with only 10% FA, at all stages of curing. The compressive strength of the non-cement specimens reached 70% that of the mortar made using Portland cement. The non-cement blended specimens with the highest compressive strength were those made using an S/FA ratio of 6:4. Results of compressive strength development were indicated that S and FA reacted with water and then with calcium hydroxide via a pozzolanic reaction to form hydration products (i.e., calcium silicate hydrate (C-S-H) and/or calcium aluminium silicate hydrate (C-A-S-H) colloids). These findings are consistent with those reported in previous studies [18,24] and it was produced produces viscous hardening behavior in the cementless composites [23].

Non-cement blended composite without alkali activator is an innovative material. For concrete design, the minimum compressive strengths of concrete for normal structural components such as walls or slabs were used as 21 MPa. For this reason, the target strength was set as 21 MPa in this study. As shown in Figure 4b, all of the mixtures reached the target strength of 21 MPa, which is suitable for normal use in civil and construction. We observed a positive correlation between the proportion of fibers and compressive strength, due perhaps to suppressed crack formation under axial loading. The inclusion of fibers in the non-cement blended composites significantly increased the compressive strength, particularly in S50-F2 and S40-F2 (30% to 40% higher than the S50) [26]. This also had indirect effects on the toughness and mechanical properties of the composites. Hydration reactions among the fibers can increase the interfacial bonding strength, resulting in higher compressive strength. These findings are also consistent with those reported in previous studies [27]. The decrease in compressive strength observed in Figure 4b when the fiber content is increased from mixes P-F2 to P-F5. This diminution may be attributed to the lump of noncontiguous fibers, which is consistent with previous study [28].

### 3.3. Tensile Strength

**Figure 5:**
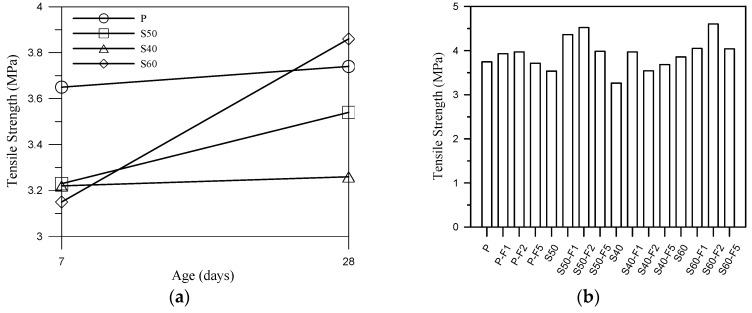
(**a**) Tensile strength development curves of select samples and (**b**) tensile strength results of all samples at 28 days.

Figure 5a illustrates the tensile strength of non-cement blended specimens and cement mortar specimens without fibers. Clearly, the increase in tensile strength in the non-cement mortar was not significant at 7 days compared to the P specimens (average 15% lower than P specimens), but quite noticeable at 28 days (the tensile strength of S60 specimen was slight higher than that of P specimens). The tensile strength of S60 was 18% higher than that of S40. We speculate that this can be attributed to the mix ratio, which allowed the S and FA to be gelatinized; however, the slowness of the reaction hindered strength development. On the basis of the previous study [29], FA can be used as a sustainable alkali activator for S to activate the alkali-activated or pozzolanic reaction. The tensile strength of the non-cement blended specimens increased with an increase in the proportion of polypropylene fibers, as shown in Figure 5b. At 28 days, the tensile strength of samples S50-F2 and S60-F2 were respectively 20% and 23% higher than that of the control specimens. This obvious increase in tensile strength can be attributed to the fibers arresting crack propagation at the micro scale. In addition, the decrease in tensile strength when the fiber content is increased from P-F2, PF5, S50-F2 and S50-F5 was also due to the lump of noncontiguous fibers in composites.

### 3.4. Drying Shrinkage

**Figure 6:**
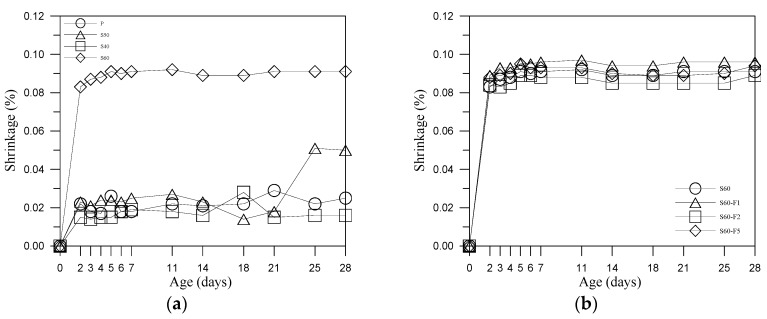
Length change results: (**a**) comparison to P, S40, S50 and S60 specimens and (**b**) comparison to S60, S60-F1, S60-F2 and S60-F5.

As shown in Figure 6a, the shrinkage development curves of S40 and S50 specimens were similar to those of P specimens. By contrast, the shrinkage development curve of specimen S60 was 4 times higher than that of cement mortar, indicating a high SO_3_ content following a rapid hydration reaction with a corresponding rapid volume expansion. Previous studies [17,18,24] have also reported on the considerable influence of SO_3_ on the strength of mortar specimens containing FA.

Our results revealed that the addition of fibers to specimen S60 (S60-F1/2/5) did not have a significant effect on shrinkage and the effect of fibers in shrinkage development curves of S40 and S50 specimens were similar to those of S60 specimens. We also found that shrinkage was largely independent of the proportion of FA. FA contains large quantities of free lime (f-CaO) and SO_3_, which are beneficial to strength development of hydrated in non-cement blended composites containing S. Note however that f-CaO and/or excessive SO_3_ can cause expansion in hardened mortar. Increasing the amount of FA could increase the number of sulphate ions in solution, thereby hindering ettringite formation [29,30]. This can have profound effects on the volume stability and strength of mortar specimens with a high proportion of FA. 

### 3.5. Absorption

**Figure 7:**
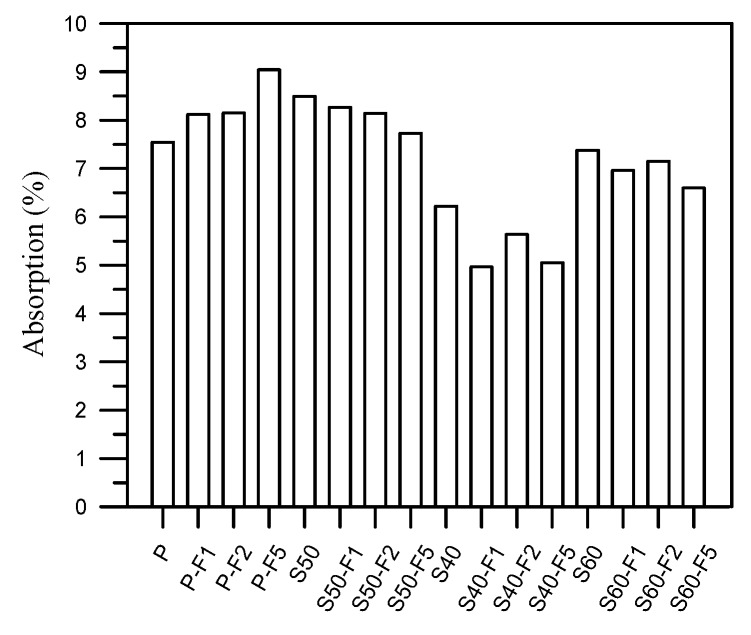
Absorption results of all samples at 56 days.

Figure 7 presents the water absorption results of all mixtures. The absorption of S40 specimens was lower than that of the P, S50 and S60. The results indicated that the absorption decreased with an increase in the proportion of S due changes in the pore structure filled by finer S. The inclusion of fine S powder improved the compactness through pozzolanic reactivity and pore filling effects. Absorption also decreased with an increase in the proportion of fibers, due to the fact that the fibers blocked the connectivity of pores, thereby making the transmission paths more complex.

### 3.6. XRD Analysis

**Figure 8:**
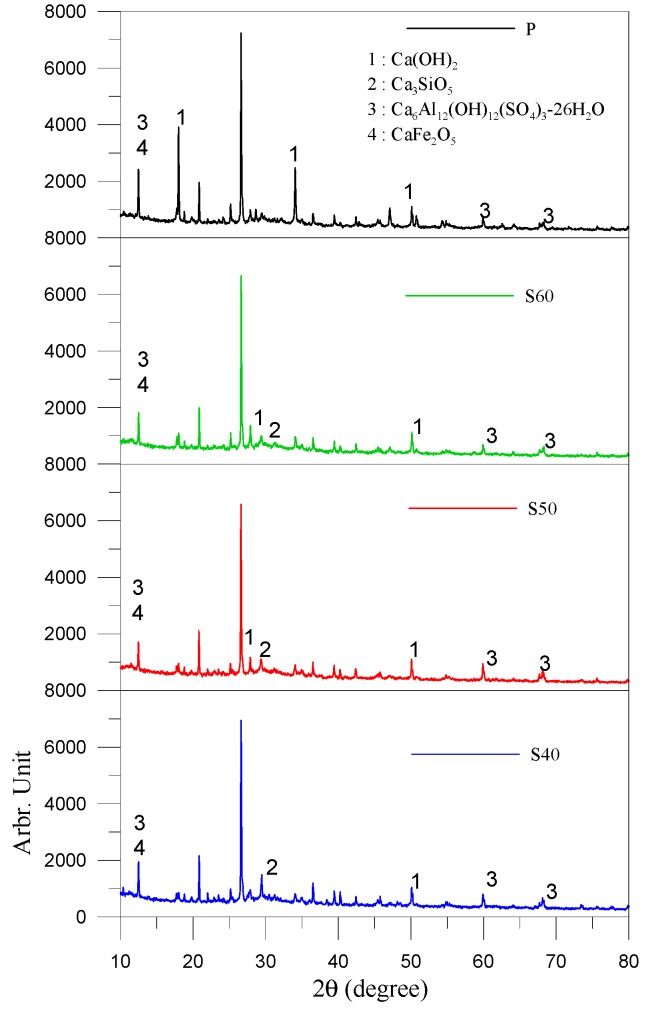
XRD patterns of various samples at 28 days.

Figure 8 presents XRD patterns of non-cement blended samples prepared using various S/FA ratios at 28 days. Hydration products appeared in various phases, including Ca(OH)_2_, Ca_3_SiO_5_, Ca_6_Al_2_(OH)_12_(SO_4_)_3_-26H_2_O and CaFe_2_O_5_. The non-cement blended specimens displayed roughly the same CaSO_4_-2H_2_O and Ca_6_Al_2_(OH)_12_(SO_4_)_3_-26H_2_O (ettringite) [31] peaks compared to the control groups. Note that the non-cement blended specimens also presented relatively small Ca(OH)_2_ peaks and slightly high Ca_3_SiO_5_ peaks compared to the P specimens. It also indicated that the hydration products of non-cement blended specimens were similar to the P specimens and almost less calcium hydroxide (Ca(OH)_2_) was found in non-cement blended specimens. It indicated that the quartz (SiO_2_) in the FA reacted with lime (CaO) and Ca(OH)_2_, resulting in the formation of C-S-H and C-A-S-H gel, which are the key factors in strength development in cementitious materials [32,33]. The FA also had higher proportions of f-CaO and CaSO_4_, which has been shown to activate cementing processes and hydration reactions [34]. It also confirmed that the strength of non-cement blended composites can be achieved to conventional cement-based materials at the age of 56 days.

### 3.7. SEM Observation

**Figure 9:**
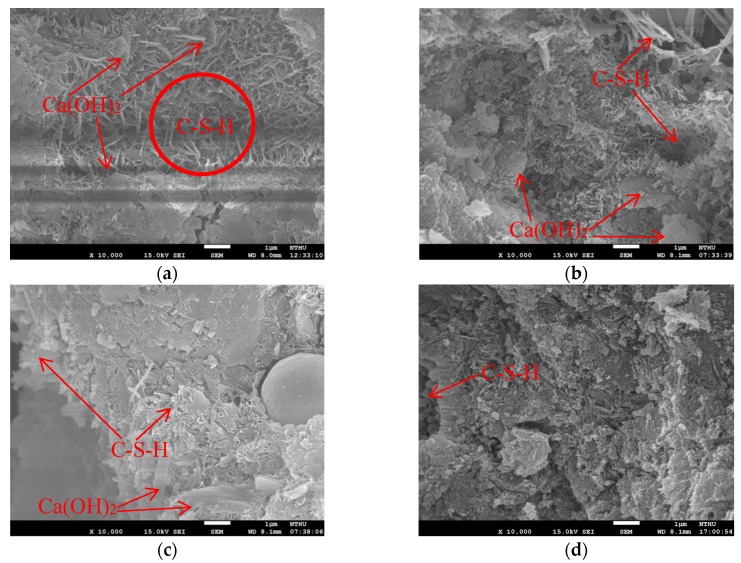
SEM photos at 28 days: (**a**) P; (**b**) S40; (**c**) S50 and (**d**) S60.

**Figure 10:**
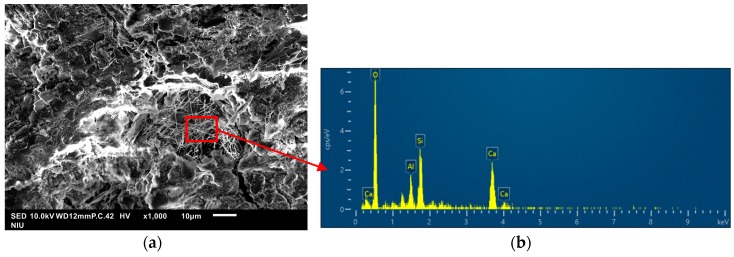
Scanning electron microscope (SEM) photos with energy dispersive analysis (EDS) analysis: (**a**) S60 specimens; (**b**) EDS results.

Figure 9 presents SEM images of various specimens under 10K× magnification at 28 days. The non-cement blended specimens presented more pronounced pore formation than did the cement specimens. C-S-H gel is the product of reactions between water and tricalcium silicate or dicalcium silicate. Approximately 50% of the cement gel was C-S-H gel, which served as the primary source of strength in the cement paste. C-S-H gel and ettringite appeared as irregular needle-like and spherical continuums, whereas Ca(OH)_2_ appears as hexagonal flakes. However, the appearance of fine exterior capillary tube spikes indicates that the C-S-H gel was covered in pores, i.e., it was not a smooth continuum. The formation of high-quality concrete depends on finer materials filling in the pores. As shown in the Figure 9 and Figure 10, most of the pores have been filled with ettringite, which gave the material a dense appearance and reduced the likelihood of infiltration by harmful substances. 

Based on the SEM images, the reaction mechanisms in non-cement blended materials comprising a mix of S and FA can be divided into two phases. In the first phase, the CaO in the FA violently reacted with water to produce Ca(OH)_2_. It was shown that increasing the proportion of FA accelerated the process of setting. Insufficient FA would slow the hydration setting speed after the S and FA mix. In the second phase, the Ca(OH)_2_ and SiO_2_ in the S and FA with reacted with SiO_2_ and Al_2_O_3_ to produce C-S-H and C-A-S-H gel [35] as shown in Figure 10. It can be consistent with previous study [18].

## 4. Conclusions

It is a feasibility to produce entirely non-cement blended materials using S with FA and FA can be as an alkali activator for S in blended composites. In this study, we produced non-cement blended materials by replacing 100% of the cement (by weight) with S and FA at ratios of 4:6, 5:5 and 6:4. Specimens made with an S:FA ratio of 6:4 achieved compressive strength of roughly 30 MPa (at 28 days), which is the 80% the strength of conventional cement-based materials (control specimens). Non-cement blended composites were used to have a proper inclusion of fiber reinforcements to conduct the engineering properties and increase its applicability for construction applications. The inclusion of 0.2% fibers in the mix further increased compressive strength to 35 MPa. It also enhanced the compactness of micro pore-structures, increased the compressive strength and tensile strength and decreased absorption and the likelihood of shrinkage. SEM images and XRD analysis revealed that the compressive strength of the non-cement blended specimens can be attributed to the formation of C-S-H and C-A-S-H gels by Ca(OH)_2_, SiO_2_, and Al_2_O_3_. Polypropylene fibers were shown to have a profound effect in reinforcing the non-cement blended materials and it has shown the feasibility of the usage of non-cement blended fiber composites in civil construction.

## Figures and Tables

**Table 1:** Chemical compositions of co-fired fly ash and GGBS.

Chemical Compositions	Co-Fired Fly Ash	GGBS
	Content, wt %	
Silicon dioxide (SiO_2_)	29.47	33.68
Aluminum oxide (Al_2_O_3_)	19.27	14.37
Ferric oxide (Fe_2_O_3_)	3.49	0.29
Calcium oxide (CaO)	35.54	40.24
Magnesium oxide (MgO)	1.82	7.83
Sulphur trioxide (SO_3_)	7.36	0.66
others	3.05	2.93

**Table 2:** Mix design of the mortar specimens produced (kg/m^3^).

Mix No.	Cement	GGBS	Co-Fired Fly Ash	Fine Aggregates	Water	Superplasticizers	Fiber
P	514	0	0	1412	282	0	0
S50	0	257	257		274.6	7.4	
S40		308	206		276.8	5.2	
S60		206	308		267.2	14.8	
P-F1	514	0	0		282	0	0.88
P-F2							1.76
P-F5							4.41
S50-F1	0	257	257		274.6	7.4	0.88
S50-F2					274.6	7.4	1.76
S50-F5					270.9	11.1	4.41
S40-F1		308	206		276.8	5.2	0.88
S40-F2					274.6	7.4	1.76
S40-F5					270.9	11.1	4.41
S60-F1		206	308		267.2	14.8	0.88
S60-F2					267.2	14.8	1.76
S60-F5					264.2	17.8	4.41

**Table 3:** Mix design of the mortar specimens for a preliminary test (kg/m^3^).

Mix No.	Cement	GGBS	Co-Fired Fly Ash	Fine Aggregates	Water
P	514	0	0	1412	282
S10	0	463	51		
S20	0	411	103		
S30	0	360	154		
S40	0	308	206		
S50	0	257	257		
S60	0	206	308		

**Table 4:** Test methods.

Test Target		Specimen Dimensions (mm)	Referenced Standard	Testing Age (days)
Fresh properties	flow test	–	ASTM C230	–
Mechanical properties	compressive strength test	50 × 50 × 50	ASTM C109	7, 28, 56
	tensile strength test	Briquet Specimens	ASTM C260 CRD-C 260-01	7, 28
	drying shrinkage test	285 × 25 × 25	ASTM C596	2~28
Permeability	water absorption test	50 × 50 × 50	ASTM C642	56
Microstructure observations	SEM observation	10 × 10 × 3	ASTM C1723	28
	XRD analysis	powders	ASTM C1365	28, 56
